# Characteristics of Kawasaki Disease Before and After the COVID-19 Pandemic in a Large Pediatric Heart Disease Center

**DOI:** 10.3389/fped.2022.895408

**Published:** 2022-05-17

**Authors:** Huan Yu, Chao Ni, Yuhan Xia, Jie Li, Biyao Hang, Cheng Han, Zhipeng Xu, Ming Luo, Xing Rong, Jinshun Zhu, Maoping Chu

**Affiliations:** ^1^Department of Pediatrics, Second Affiliated Hospital and Yuying Children's Hospital of Wenzhou Medical University, Wenzhou, China; ^2^Department of Pediatrics, Guangyuan Central Hospital, Guangyuan, China

**Keywords:** Kawasaki, COVID-19, age, hospitalization, albumin, CALs

## Abstract

**Background:**

Kawasaki disease (KD) is an acute febrile systemic vasculitis of unknown etiology. After the pandemic of coronavirus disease 2019 (COVID-19), some children infected by severe acute respiratory syndrome coronavirus 2 (SARS-CoV-2) showed clinical symptoms similar to KD, indicating a close relationship between KD and SARS-CoV-2. Therefore, we designed this retrospective study to analyze the characteristics of KD patients before and after the COVID-19 pandemic.

**Methods:**

We retrospectively collected demographic and laboratory data of KD patients in Yuying Children's Hospital of Wenzhou Medical University from 1 January 2015 to 31 December 2020. Yuying Children's Hospital of Wenzhou Medical University is located in eastern China and is the largest pediatric heart disease center in the region, which includes a population of nearly 10 million. We studied the characteristics of KD patients and analyzed the changes in these characteristics before and after the emergence of SARS-CoV-2 in this area.

**Results:**

The analysis revealed the following novel features: (1) Under the influence of the COVID-19 pandemic, the onset age of Kawasaki disease became younger. (2) After the occurrence of COVID-19, the hospitalization days of KD patients were shorter than before the pandemic. (3) After the occurrence of COVID-19, the albumin of KD patients was higher than before the pandemic. (4) The COVID-19 pandemic did not have a significant effect on the incidence of coronary artery lesions (CALs) in Kawasaki disease.

**Conclusion:**

After the COVID-19 outbreak, the characteristics of KD patients showed a younger trend of age, shorter hospitalization days and higher levels of albumin, but the incidence of CALs did not change significantly.

## Highlights

- The Characteristics of KD Before and After the COVID-19 Pandemic Were first Discussed in China.
- Our Results Provide a Reference for the Characteristics of KD in Other COVID-19 Pandemic Areas.
- Clinical and Laboratory Data of KD Patients Were Collected in the Largest Pediatric Heart Disease Center in a Region With a Population of 10 Million, Which can Represent the Characteristics of KD in a Large Population Area.

## Introduction

Kawasaki disease (KD) is a small and medium-sized blood vessel vasculitis that usually affects children under 5 years of age (1). KD was first proposed in 1967, but the causes remain unknown (2). KD tends to involve the coronary artery, leading to coronary artery lesions (CALs) or even coronary artery aneurysms (CAAs) (3).

The outbreak of coronavirus disease 2019 (COVID-19) affects a large number of people, including children, and poses severe challenges to the world (4, 5). Britain, the United States (6), Italy (7), France (8, 9) and other countries (10) have reported cases of children infected with SARS-CoV-2 with symptoms similar to KD. This phenomenon has been called pediatric multisystem inflammatory syndrome temporally associated with SARS-CoV-2 (PIMS-TS) in the UK and multisystem inflammatory syndrome in children (MIS-C) in the USA (11). Studies have compared the characteristics of SARS-CoV-2-positive and SARS-CoV-2-negative children among patients with KD, hoping to provide a better explanation for the disease (12). We are also interested in this topic, but since no SARS-CoV-2-positive children with KD have been found in this area due to strict prevention and control methods, we focused on whether the pandemic has brought changes to the characteristics of KD patients.

China, as a country with a large population, brought the COVID-19 pandemic under control soon after its outbreak. However, at the same time, the pandemic greatly changed the living and social habits of Chinese people, especially from January to March 2020, when the pandemic was the most severe. During this period, China adopted measures such as blockading cities and promoting less travel to cut the spread of the pandemic through social isolation (e.g., every family in the city could assign one family member to go out to purchase necessities every 2 days) (13, 14). This policy was very successful in the acute stage of the pandemic (15). Since April 2020, the pandemic situation has gradually improved, and moderate social distancing and mask-wearing have become common in daily life. We were curious whether the outbreak of COVID-19 and the changes in prevention and control policies had an impact on the characteristics of KD patients, and we designed this retrospective study accordingly.

Yuying Children's Hospital of Wenzhou Medical University is located in eastern China; it is the largest pediatric heart center in the region and can represent the characteristics of KD in a large population area. Therefore, we retrospectively analyzed the characteristics of KD patients before and after the outbreak of COVID-19 and found interesting phenomena.

## Methods

### Patient Enrollment

This study was approved by Yuying Children's Hospital of Wenzhou Medical University. Patients who met the diagnostic criteria of Kawasaki disease from 2015 to 2020 based on the 2017 American Heart Association (AHA) guidelines were included in the study. KD was diagnosed in the presence of fever together with at least 4 of the 5 following principal clinical features: (1) erythema and cracking of lips, strawberry tongue, and/or erythema of oral and pharyngeal mucosa; (2) bilateral bulbar conjunctival injection without exudate; (3) rash: maculopapular, diffuse erythroderma, or erythema; (4) multiforme-like erythema and edema of the hands and feet in the acute phase and/or periungual desquamation in the subacute phase; (5) cervical lymphadenopathy. Patients who lacked the full clinical features of classic KD were often evaluated for incomplete KD. If coronary artery abnormalities were detected, the diagnosis of KD was considered confirmed in most cases. KD patients who were not treated with intravenous immunoglobulin (IVIG), were not in the acute stage of KD or had an unclear diagnosis were excluded. In addition, all patients enrolled after the outbreak of COVID-19 were tested with a real-time RT–PCR assay, and all the results were negative. Meanwhile, the epidemiological investigation showed that the children had no history of close contact with individuals with COVID-19.

### Study Design

For the purpose of our study, we retrospectively compared the clinical and laboratory data of KD patients between 2019 and 2020 and obtained interesting results. To verify the findings, KD patients from 1 January 2015 to 31 December 2019 were compared with KD patients in 2020.

Meanwhile, the control and prevention of the COVID-19 pandemic in China was the most severe from January to March 2020 and gradually relaxed after April 2020, and we compared KD patients between the two different periods. The above analysis revealed an interesting result of onset ages among KD patients. To better illustrate this finding, we divided KD patients according to age and conducted further statistical analyses.

### Demographic Data and Laboratory Tests of KD Patients

The diagnosis of all patients was determined by experienced pediatric cardiologists, including the identification of incomplete Kawasaki disease (IKD) and intravenous immunoglobulin resistance (IVIG-R). The laboratory tests were carried out by the Laboratory Department of Yuying Children's Hospital of Wenzhou Medical University and included hemoglobin (HB), platelet (PLT), white blood cell (WBC), neutrophil (N), lymphocyte (L), eosinophil (E), hematocrit (HCT), c-reactive protein (CRP), erythrocyte sedimentation rate (ESR), sodium (Na), chlorine (Cl), potassium (K), calcium (Ca), magnesium (Mg), phosphorus (P), aspartate aminotransferase (ALT), alanine aminotransferase (AST), albumin (ALB), gamma glutamate transferase (GGT), total bilirubin (TBIL), direct bilirubin (DBIL), and indirect bilirubin (IBIL) levels. The echocardiography of the patients was examined by doctors in the ultrasound department of the hospital. The standard definition of a CAL is as follows: (1) coronary artery diameter > 2.5 mm for children aged <3 years, > 3 mm for children aged 3–9 years, and > 3.5 mm for children aged > 9 years; (2) internal diameter of a segment measuring ≥1.5 times that of an adjacent segment; and (3) clearly irregular lumen (16).

### Follow-Up Care

Children who did not revisit after discharge were defined as missing cases. The follow-up period was from 1 to 6 months. The follow-up data of pediatric outpatients were collected by pediatric cardiovascular doctors.

### Statistical Methods

The classified variables were analyzed by the chi-square test. The continuous variables were analyzed by *t* test or non-parametric test according to the normality test of the data. *P* < 0.05 indicated a statistically significant difference.

### Patient and Public Involvement

Patients and the public were not involved in the design, conduct, reporting, or dissemination plan of this research.

## Results

### Clinical Data and Laboratory Test Comparisons Between 2019 and 2020 KD Patients

The results indicated that the onset age of KD patients in 2020 was lower than that in 2019, and the hospitalization days were shorter. There were no significant differences in sex, IKD or IVIG-R incidence between the two groups. The laboratory test results revealed that N, ESR, Mg and GGT in KD patients in 2020 were lower than those in 2019, while the levels of L, Cl and ALB were higher. There were no significant differences in other indices. The results are presented in Table 1.

**Table 1:** Demographic and clinical characteristics.

**Items**	**2020**	**2019**	***P***
Male (%)	138 (62.2%)	228 (64%)	0.658
Age (month)	25.55 ± 25.22	29.14 ± 22.27	0.002^*^
IKD (%)	80 (36%)	103 (28.9%)	0.081
IVIG-R (%)	13 (5.9%)	21 (5.9%)	1
Hosday (days)	7.06 ± 3.28	7.44 ± 3.19	0.024^*^
HB (g/L)	111.87 ± 13.14	112.66 ± 9.68	0.682
PLT (×10^12^)	367.30 ± 151.37	371.21 ± 127.63	0.335
WBC (×10^12^)	15.93 ± 9.72	15.83 ± 6.29	0.315
N (×10^12^)	10.08 ± 4.91	10.92 ± 5.36	0.032^*^
L (×10^12^)	3.76 ± 2.04	3.43 ± 2.12	0.023^*^
E (×10^12^)	0.44 ± 0.67	0.39 ± 0.42	0.253
HCT (%)	0.35 ± 0.27	0.34 ± 0.28	0.69
CRP (mg/dl)	83.85 ± 53.58	91.68 ± 59.65	0.179
ESR (mm/h)	38.84 ± 17.34	45.68 ± 18.49	<0.001^*^
Na (mmol/L)	135.51 ± 2.39	135.26 ± 2.42	0.237
K (mmol/L)	4.41 ± 0.59	4.37 ± 0.51	0.103
Cl (mmol/L)	101.8 ± 2.64	101.19 ± 2.85	0.012^*^
Ca (mmol/L)	2.31 ± 0.14	3.35 ± 0.13	<0.001^*^
Mg (mmol/L)	0.88 ± 0.12	0.90 ± 0.09	0.007^*^
P (mmol/L)	1.39 ± 0.27	1.40 ± 0.27	0.926
ALT (U/L)	71.83 ± 111.92	80.05 ± 113.59	0.062
AST (U/L)	73.30 ± 117.17	66.34 ± 118.77	0.197
ALB (g/L)	41.00 ± 5.66	39.67 ± 4.02	0.001^*^
GGT (U/L)	72.42 ± 99.44	76.45 ± 89.24	0.06
TBIL (umol/L)	10.66 ± 11.36	10.83 ± 14.25	0.201
DBIL (umol/L)	3.96 ± 6.68	4.09 ± 8.00	0.493
IBIL (umol/L)	5.43 ± 4.26	5.39 ± 4.81	0.932

*IKD, incomplete Kawasaki disease; IVIG-R, intravenous immunoglobulin-resistance; HB, hemoglobin; PLT, platelets; WBC, white blood cell; N, neutrophils; L, lymphocyte; E, eosinophil; HCT, hematocrit; CRP, c-reactive protein; ESR, erythrocyte sedimentation rate; Na, sodium; K, potassium; Cl, chlorine; Ca, calcium; Mg, magnesium; P, phosphorus; ALT, aspartate aminotransferase; AST, alanine aminotransferase; ALB, albumin; GGT, gamma glutamate transferase; TBIL, total bilirubin; DBIL, direct bilirubin; IBIL, indirect bilirubin*.

* *p < 0.05*.

### Comparisons of Data Between KD Patients in 2015–2019 and in 2020

Consistent with the above results, a lower onset age and shorter hospitalization days of KD patients were found in 2020. A higher incidence of IKD was revealed in 2020 KD patients. There was no significant difference in sex or the incidence of IVIG-R between the two groups. The laboratory test results showed that after the outbreak of COVID-19, the levels of WBC, N and Mg in KD patients were lower than before the pandemic, but the levels of K and Cl increased; at the same time, AST, ALB, and TBIL increased, and ALT and GGT decreased. No significant differences were found in other indices. The results are presented in Table 2.

**Table 2:** Demographic and clinical characteristics.

**Items**	**2020**	**2015–2019**	***P***
Male (%)	138 (62.2%)	945 (64.3%)	0.549
Age (month)	25.69 ± 25.59	27.41 ± 22.37	0.022^*^
IKD (%)	80 (36%)	387 (26.3%)	0.004^*^
IVIG-R (%)	13 (5.9%)	91 (6.1%)	1
Hosday (days)	7.12 ± 3.32	8.34 ± 3.36	<0.001^*^
HB (g/L)	111.92 ± 13.28	112.11 ± 10.38	0.338
PLT (×10^12^)	368.76 ± 152.85	378.84 ± 129.19	0.086
WBC (×10^12^)	15.42 ± 7.00	15.93 ± 5.77	0.033^*^
N (×10^12^)	9.96 ± 4.92	10.86 ± 5.02	0.003^*^
L (×10^12^)	3.81 ± 2.04	3.71 ± 2.15	0.288
E (×10^12^)	0.44 ± 0.69	0.39 ± 0.45	0.134
HCT (%)	0.35 ± 0.27	0.34 ± 0.31	0.45
CRP (mg/dl)	81.40 ± 52.22	85.18 ± 56.88	0.481
ESR (mm/h)	38.64 ± 17.53	37.4 ± 15.25	0.185
Na (mmol/L)	135.54 ± 2.37	135.60 ± 2.57	0.76
K (mmol/L)	4.42 ± 0.58	4.33 ± 0.55	0.015^*^
Cl (mmol/L)	101.90 ± 2.62	101.05 ± 2.84	<0.001^*^
Ca (mmol/L)	2.31 ± 0.14	2.32 ± 0.13	0.311
Mg (mmol/L)	0.89 ± 0.12	0.91 ± 0.10	<0.001^*^
P (mmol/L)	1.40 ± 0.27	1.39 ± 0.27	0.603
ALT (U/L)	70.68 ± 111.00	77.12 ± 106.96	0.01^*^
AST (U/L)	73.60 ± 119.15	59.21 ± 94.58	0.021^*^
ALB (g/L)	40.91 ± 5.16	39.78 ± 4.02	<0.001^*^
GGT (U/L)	70.76 ± 97.75	82.25 ± 96.99	0.005^*^
TBIL (umol/L)	10.48 ± 11.28	9.51 ± 11.56	0.013^*^
DBIL (umol/L)	3.76 ± 6.45	3.63 ± 6.65	0.138
IBIL (umol/L)	5.29 ± 4.22	5.16 ± 4.78	0.658

*IKD, incomplete Kawasaki disease; IVIG-R, intravenous immunoglobulin-resistance; HB, hemoglobin; PLT, platelets; WBC, white blood cell; N, neutrophils; L, lymphocyte; E, eosinophil; HCT, hematocrit; CRP, c-reactive protein; ESR, erythrocyte sedimentation rate; Na, sodium; K, potassium; Cl, chlorine; Ca, calcium; Mg, magnesium; P, phosphorus; ALT, aspartate aminotransferase; AST, alanine aminotransferase; ALB, albumin; GGT, gamma glutamate transferase; TBIL, total bilirubin; DBIL, direct bilirubin; IBIL, indirect bilirubin*.

* *p < 0.05*.

### Comparison of KD Patients Between 1 January to 31 March 2020 and 1 April to 31 December 2020

The onset age of KD patients after 1 April 2020 was lower than that between 1 January and 31 March 2020. The incidence of IKD was lower, the hospitalization days were shorter, and the levels of HB, WBC, N, HCT and GGT were higher, but the levels of Na, Cl and Ca were lower. The detailed statistical results are shown in Table 3.

**Table 3:** Demographic and clinical characteristics.

**Items**	**2020.1.1-3.31**	**2020.4.1-12.31**	***P***
Male (%)	38 (56.7%)	100 (64.5%)	0.294
Age (month)	33.48 ± 30.30	22.32 ± 22.56	0.003^*^
IKD (%)	16 (23.9%)	64 (41.3%)	0.015^*^
IVIG-R (%)	6 (9.0%)	7 (4.5%)	0.217
Hosday (days)	6.37 ± 3.05	7.44 ± 3.38	0.001^*^
HB (g/L)	115.33 ± 11.34	110.44 ± 13.82	0.008^*^
PLT (×10^12^)	349.10 ± 118.41	377.31 ± 165.23	0.492
WBC (×10^12^)	17.02 ± 8.94	14.72 ± 5.86	0.029^*^
N (×10^12^)	11.11 ± 4.97	9.46 ± 4.83	0.004^*^
L (×10^12^)	3.76 ± 2.04	3.71 ± 2.15	0.288
E (×10^12^)	0.44 ± 0.67	0.39 ± 0.45	0.134
HCT (%)	0.40 ± 0.49	0.33 ± 0.03	0.044^*^
CRP (mg/dl)	85.09 ± 45.73	79.8 ± 54.87	0.187
ESR (mm/h)	37.38 ± 14.19	39.23 ± 18.9	0.459
Na (mmol/L)	135.12 ± 1.86	135.73 ± 2.55	0.024^*^
K (mmol/L)	4.35 ± 0.48	4.45 ± 0.62	0.16
Cl (mmol/L)	101.00 ± 2.26	102.30 ± 2.68	0.001^*^
Ca (mmol/L)	2.28 ± 0.13	2.33 ± 0.14	0.017^*^
Mg (mmol/L)	0.88 ± 0.14	0.89 ± 0.11	0.544
P (mmol/L)	1.37 ± 0.24	1.42 ± 0.28	0.187
ALT (U/L)	82.39 ± 146.86	65.55 ± 91.11	0.98
AST (U/L)	84.16 ± 166.85	68.48 ± 87.57	0.858
ALB (g/L)	41.06 ± 4.78	40.84 ± 5.33	0.389
GGT (U/L)	76.57 ± 111.15	67.80 ± 90.58	0.005^*^
TBIL (umol/L)	9.80 ± 8.51	10.78 ± 12.32	0.804
DBIL (umol/L)	3.54 ± 5.18	3.87 ± 7.00	0.881
IBIL (umol/L)	5.37 ± 3.64	5.26 ± 4.49	0.37

*IKD, incomplete Kawasaki disease; IVIG-R, intravenous immunoglobulin-resistance; HB, hemoglobin; PLT, platelets; WBC, white blood cell; N, neutrophils; L, lymphocyte; E, eosinophil; HCT, hematocrit; CRP, c-reactive protein; ESR, erythrocyte sedimentation rate; Na, sodium; K, potassium; Cl, chlorine; Ca, calcium; Mg, magnesium; P, phosphorus; ALT, aspartate aminotransferase; AST, alanine aminotransferase; ALB, albumin; GGT, gamma glutamate transferase; TBIL, total bilirubin; DBIL, direct bilirubin; IBIL, indirect bilirubin*.

* *p < 0.05*.

### Comparison of CAL Incidence

We analyzed the incidence of CALs between 2015–2019 and 2020, and no significant difference was found. The same analysis was conducted between 2019 and 2020 and the conclusion was consistent, as shown in Table 4.

**Table 4:** The comparision of CAL incidence.

	**2015-2019**	**2020**	**2019**
CAL before treatment	470 (32.0%)	61 (27.5%)	105 (29.5%)
	*P =* 0.188		
		*P =* 0.637	
CAL after treatment	454 (30.9%)	59 (26.6%)	99 (27.8%)
	*P =* 0.21		
		*P =* 0.774	

CAL was defined as mentioned in the article. CAL before treatment means that CAL was calculated before treatment with IVIG to the end of follow-up. CAL after treatment means that CAL was calculated after treatment with IVIG to the end of follow-up. Patients who had a CAL at any time during the study period were counted. *P < 0.05.

### Changes in the Characteristics of Patients With Kawasaki Disease After COVID-19

**Figure 1:**
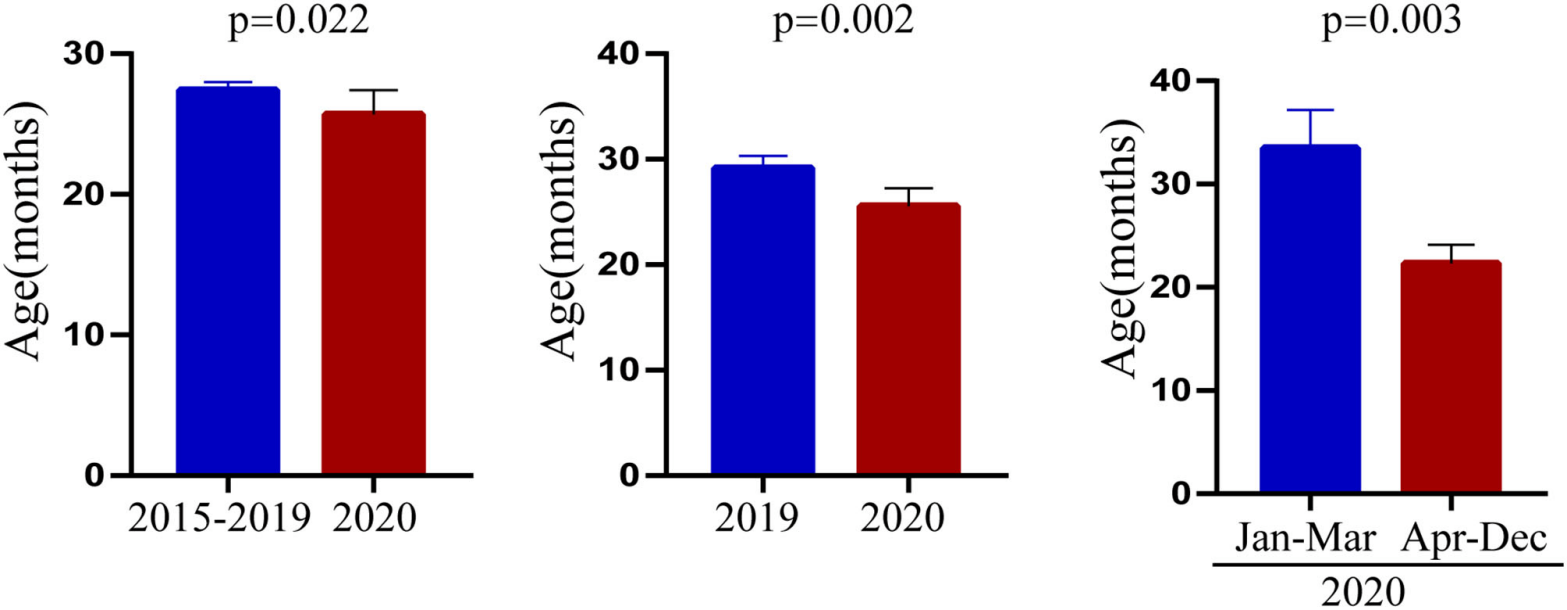
Characteristics of ages before and after the COVID-19 pandemic.

**Figure 2:**
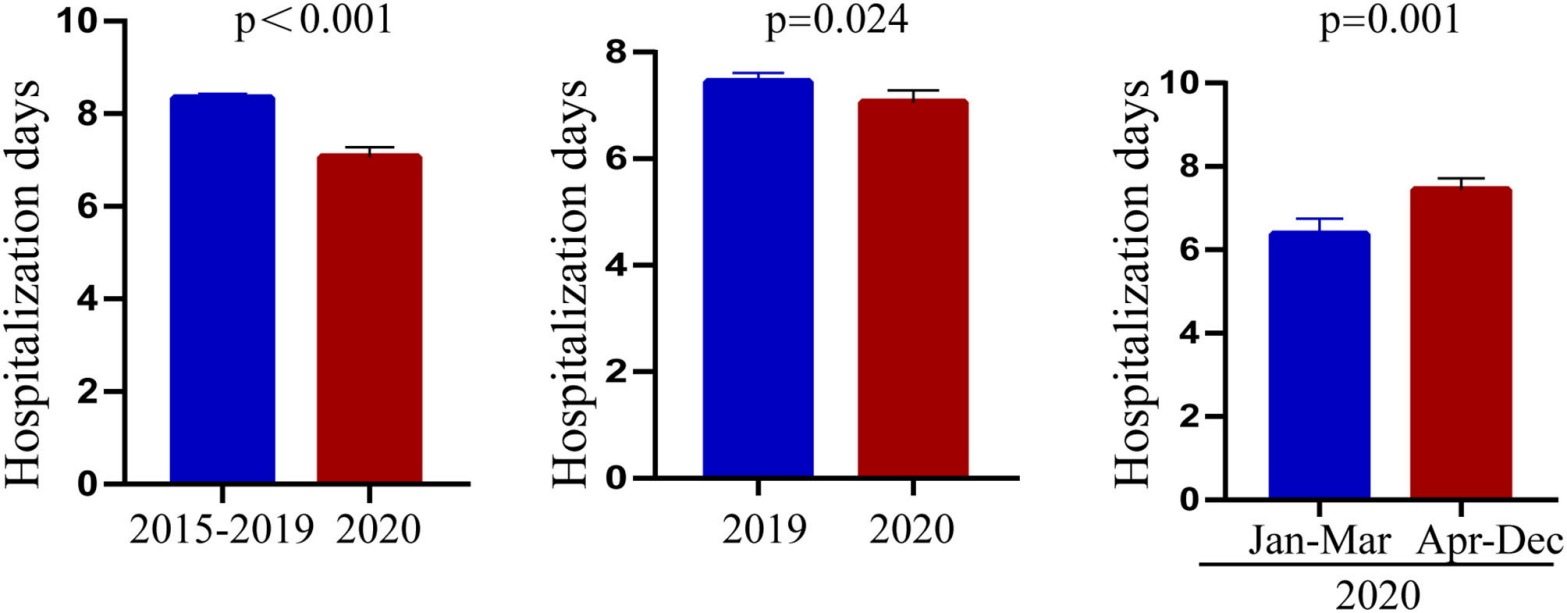
Characteristics of hospitalization days before and after the COVID-19 pandemic.

**Figure 3:**
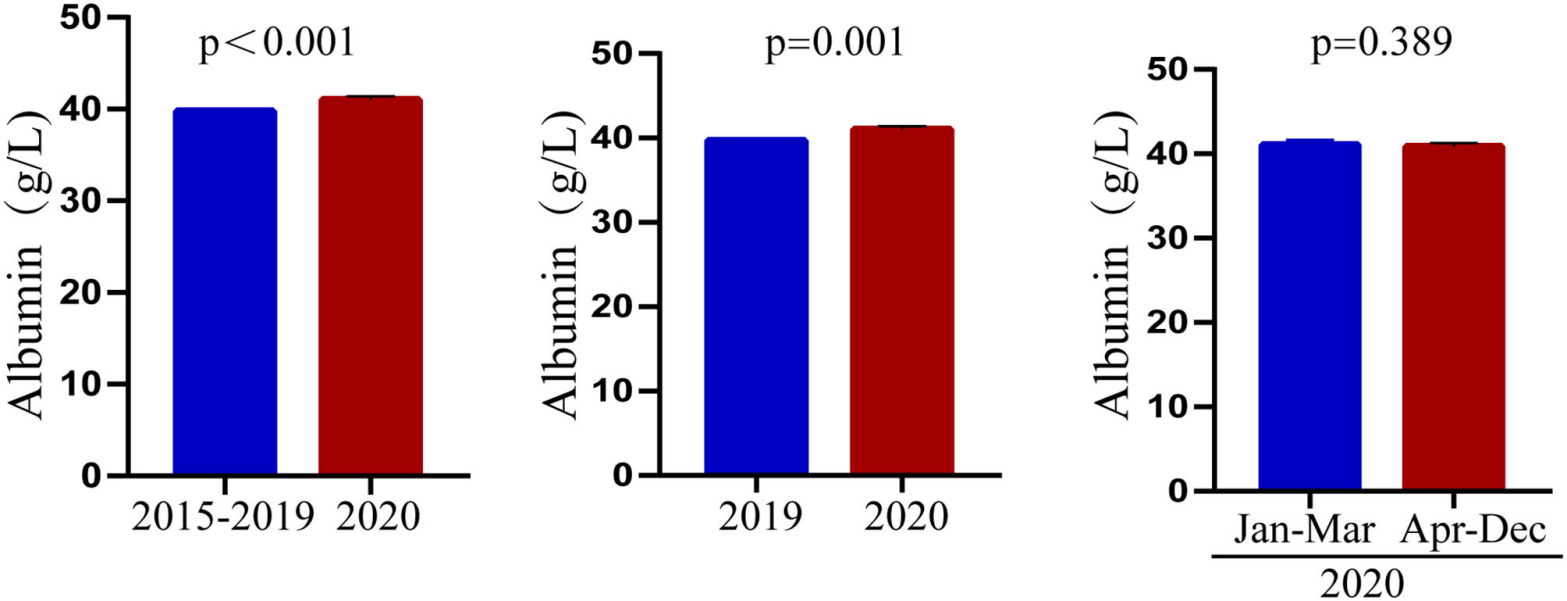
Characteristics of albumin before and after the COVID-19 pandemic.

Through the above statistical analysis, we found that after the outbreak of COVID-19, there was an obvious younger trend in the onset age of KD, the days of hospitalization were shorter, and the level of albumin was significantly higher than before the pandemic. To better illustrate these changes, the results are presented in Figures 1–3.

### Future Analysis for Age Stratification

Among the results, the younger trend of onset age is the most worthy of our attention. We further divided patients into three groups by age, ≤ 1 year, 1–5 years and > 5 years, and performed a statistical analysis.

The comparison of age stratification between 2015 to 2019 and 2020 suggested that the proportion of younger KD patients in the 2020 group was higher than that in the 2015–2019 group, and the difference was statistically significant (Table 5). The same results were obtained in the comparisons of the 2019 and 2020 groups (Table 6). We further compared the same age stratification between 1 January to 31 March 2020 and 1 April to 31 December 2020 and obtained the same results (Table 7).

**Table 5:** The comparision between different ages.

**Age**	**2015–2019**	**2020**	***P***
≤1y	392 (26.7%)	79 (35.6%)	0.022^*^
1–5y	939 (63.9%)	125 (56.3%)	
>5y	139 (9.5%)	18 (8.1%)	

*Comparison between different ages among patients enrolled from 2015 to 2019 and patients admitted in 2020*.

* *P < 0.05*.

**Table 6:** The comparision between different ages.

**Age**	**2019**	**2020**	***P***
≤1y	79 (22.2%)	79 (35.6%)	0.002^*^
1–5y	240 (67.4%)	125 (56.3%)	
>5y	37 (10.4%)	18 (8.1%)	

*Comparison between different ages among patients enrolled in 2019 and patients admitted in 2020*.

* *P < 0.05*.

**Table 7:** The comparision between different ages.

**Age**	**2020.1-3**	**2020.4-12**	***P***
≤1y	19 (28.4%)	60 (38.7%)	0.032^*^
1–5y	38 (56.7%)	87 (56.1%)	
>5y	10 (14.9%)	8 (5.2%)	

*Comparison between different ages among patients enrolled from January to March 2020 and patients admitted from April to December 2020*.

* *P < 0.05*.

## Discussion

KD is an acute vasculitis disease that mainly involves small and medium-sized blood vessels, especially in children under 5 years of age (17). The worst damage is the involvement of the coronary artery, especially the formation of coronary artery aneurysms (18). IVIG treatment can reduce the incidence of CAL (19). In the past, Kawasaki disease was considered to be a self-limiting disease, but in recent years, an increasing number of studies support that its vascular injury exists after the acute stage and is even related to vascular stenosis, occlusion and acute sudden death in adulthood (20).

After the outbreak of COVID-19, the emergence of PIMS-TS/MIS-C drew increased attention to Kawasaki disease (21, 22). We collected cases of KD from 2015 to 2020 in a children's hospital in southeastern China and compared the clinical data and laboratory tests before and after the outbreak of the pandemic.

As shown in the results, several interesting changes attracted our attention. First, under the influence of the pandemic, the onset age of KD patients decreased. Second, the level of albumin was not affected by the pandemic but showed an upward trend, indicating that the nutritional level of KD patients had not been affected. Third, the hospitalization days of KD patients were shorter, which might reflect that the hospital shortened the hospitalization days as much as possible to reduce the risk of infection after the pandemic, although the incidence of CALs was not affected.

Regarding the age of onset, to verify the reliability of the results, patients were divided into three age groups of ≤ 1 year, 1–5 years and >5 years. There were significant differences among the three groups, and the trend of younger age mainly occurred after the acute phase rather than in the acute phase. This result was very interesting and might be related to the changes in the social style of young children under the prevention and control of the pandemic in southeastern China. Since the outbreak of the pandemic, Wenzhou was closed once in the acute period, and the main activity places for infants and young children changed from outdoor to indoor, a change that mainly occurred in children who could walk and were over 1 year of age. Studies have shown that social isolation reduced the incidence of common infectious diseases in children during the pandemic (23), which supports our theory. Wearing masks while going out reduced the risks of respiratory tract infection in children to some extent, which might be the reason for partly reducing the risk of KD in children over 1 year of age. Studies have shown that protective measures such as wearing masks and washing hands frequently reduce the incidence of respiratory tract infection (24, 25), which also supports our theory. Considering the characteristics of the COVID-19 pandemic and the prevention and control measures in China, we believe that this may be one of the reasons for the younger onset trend of KD, which in turn partially proves the link between KD and respiratory tract infection.

A national study in Korea showed that during the period of wearing masks and social isolation, the incidence of KD decreased, and this downward trend mainly occurred in people aged 0–4 and 5–9, but no decline was observed in people aged 10–19 (26). In our study, we made a more accurate classification of age according to the characteristics of KD. The results showed that there was indeed a difference in KD age stratification before and after the pandemic, mainly manifested by the decrease in patients aged 1–5 years and the increase in patients under 1 year of age. However, in the acute phase of the outbreak, the number of patients over 5 years of age decreased, and the number of patients under 1 year of age increased. In addition, a study in Japan showed that during periods of social alienation, the decline in older KD children was greater than that in KD infants (<1 year), resulting in a significant difference in the proportion of infants between 2020 and 2017–2019 (27), which was consistent with our results. This also indicates that the incidence of Kawasaki disease is closely related to infection.

There are other issues to consider. Does this trend exist in other COVID-19 pandemic areas? How long will this trend last? Will this trend be reversed by pandemic control? These are all issues that we should focus on in the future.

There are some shortcomings in our research, such as a small sample size and a single research center. Our conclusions need to be further verified by multicenter and large sample studies.

## Data Availability Statement

The raw data supporting the conclusions of this article will be made available by the authors, without undue reservation.

## Ethics Statement

The studies involving human participants were reviewed and approved by the Ethical Decision Committee of the Research Administration at Second Affiliated Hospital and Yuying Children's Hospital of Wenzhou Medical University. Written informed consent to participate in this study was provided by the participants' legal guardian/next of kin.

## Author Contributions

HY was responsible for data statistics and writing paper. CN, YX, JL, BH, CH, ZX, ML, and XR collected data. JZ and MC provided resources and designed the study. All authors contributed to the article and approved the submitted version.

## Funding

This work was supported by the National Natural Science Foundation of China (grant nos. 81970435 and 82100523).

## Conflict of Interest

The authors declare that the research was conducted in the absence of any commercial or financial relationships that could be construed as a potential conflict of interest.

## Publisher's Note

All claims expressed in this article are solely those of the authors and do not necessarily represent those of their affiliated organizations, or those of the publisher, the editors and the reviewers. Any product that may be evaluated in this article, or claim that may be made by its manufacturer, is not guaranteed or endorsed by the publisher.
